# Supplementary material for: Robust quantum valley Hall effect for vortices in an interacting bosonic quantum fluid
Source: Nat Commun. 2018 Sep 28;9:3991. doi: 10.1038/s41467-018-06520-7 (PMC6162209; doi:10.1038/s41467-018-06520-7)
Supplement: Supplementary file 1 — Supplementary Information [file 41467_2018_6520_MOESM1_ESM.pdf]

Supplementary Notes

Supplementary Figures

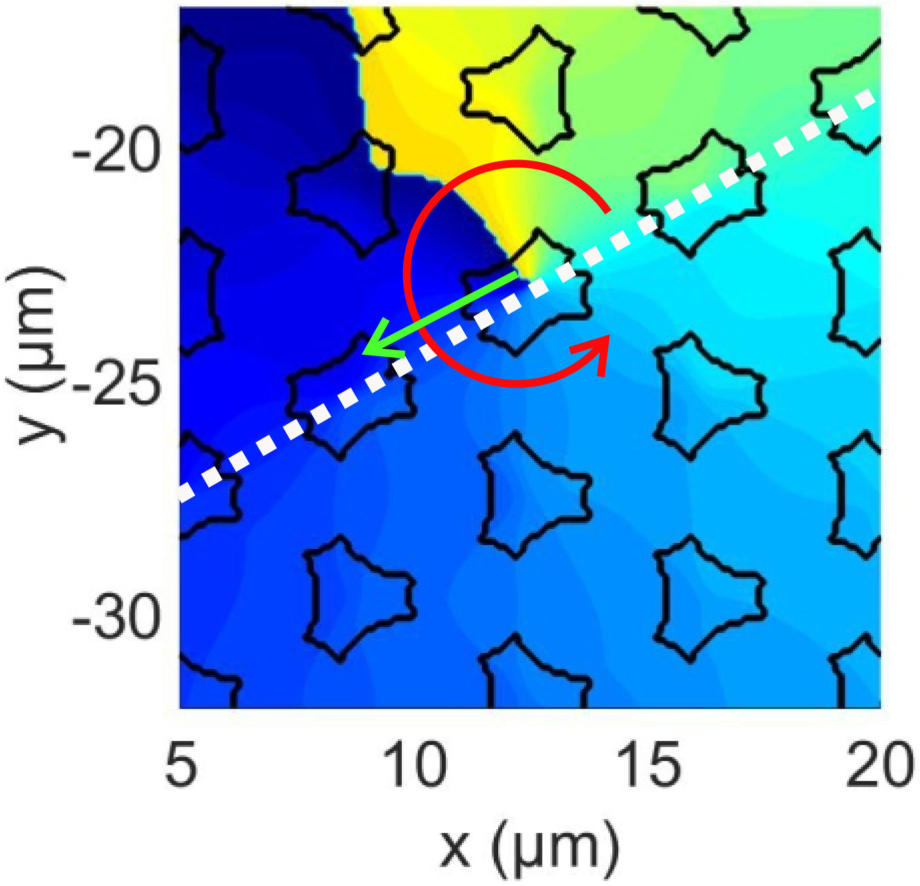

Supplementary Figure 1: Contour plot of the potential (black line) and the phase of the vortex (in color). Red arrow shows the rotation direction, green arrow shows the propagation direction of the vortex.

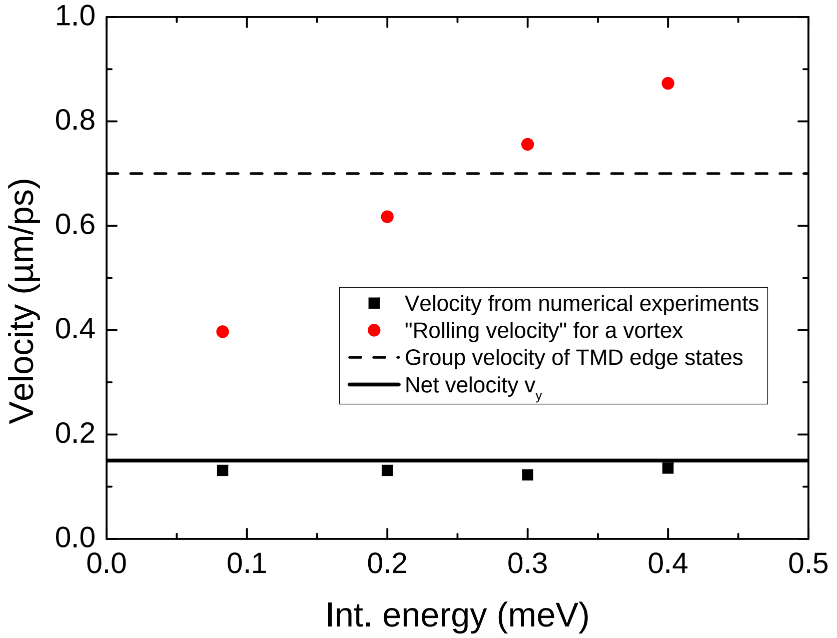

Supplementary Figure 2: Vortex velocity from numerical calculations and its estimation by different models.

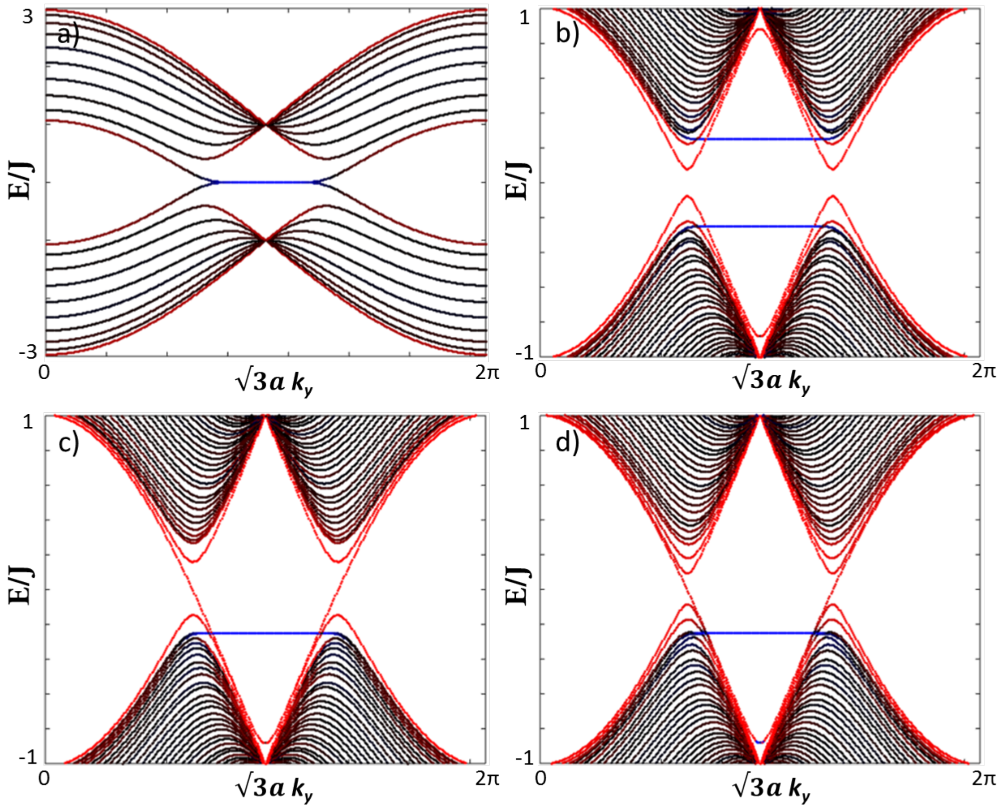

Supplementary Figure 3: Tight-binding dispersions of zigzag ribbons. (a) 10 zigzag chains of regular honeycomb, (b) wide interface (10 unstaggered chains) between two staggered honeycomb with same staggering potential, (c)-(d) Interface between two staggered honeycomb with opposite staggering potential constituted of 10 and 20 unstaggered chains respectively ($\Delta = \pm 0.25J$)

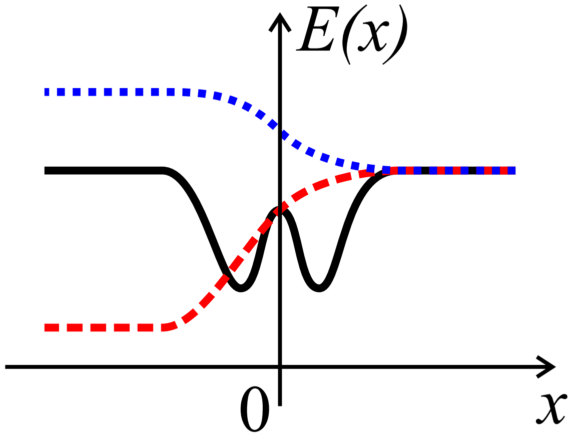

Supplementary Figure 4: Energy of the vortex as a function of position: two-domain configuration (black solid), single-domain flake surrounded by vacuum (red dashed), single-domain flake surrounded by a condensate (blue dotted).

Supplementary Note 1. Vortex-valley coupling

The calculation of the Fourier transform $\widetilde{\psi}(\mathbf{k})$ from the main text is carried out as follows. In the TB approximation, $\psi(\mathbf{r})$ is defined only in discrete points in space, and the integration is replaced by summation. Studying the core only, we take into account only the 3 atoms of the A type of the central hexagon.

This gives the following sum:

$$\begin{aligned}\widetilde{\psi}_p(\mathbf{k}) &= e^{i(0-(k_x,k_y)(0,0))} + e^{i\left(\frac{2\pi}{3}p-(k_x,k_y)\left(\frac{3a}{2},\frac{a\sqrt{3}}{2}\right)\right)} \\ &+ e^{i\left(\frac{4\pi}{3}p-(k_x,k_y)\left(0,a\sqrt{3}\right)\right)}\end{aligned} \quad (1)$$

where $p=\pm1$ is the vortex winding. This expression can be rewritten as

$$\begin{aligned}\widetilde{\psi}_p(\mathbf{k}) &= 1 + e^{i\left(\frac{2\pi}{3}p-\frac{3}{2}ak_x-\frac{\sqrt{3}}{2}ak_y\right)} \\ &+ e^{i\left(\frac{4\pi}{3}p-\sqrt{3}ak_y\right)}\end{aligned} \quad (2)$$

To simplify the expressions, let us define the arguments of the two exponents as separate variables:

$$\eta_p = \frac{2\pi}{3}p - \frac{3}{2}ak_x - \frac{\sqrt{3}}{2}ak_y \quad (3)$$

$$\zeta_p = \frac{4\pi}{3}p - \sqrt{3}ak_y \quad (4)$$

We can then find the position of the maximal probability density in the reciprocal space $|\psi(\mathbf{k})|^2$, which writes (by separating the real and imaginary parts):

$$\begin{aligned}\left|\widetilde{\psi}_p(\mathbf{k})\right|^2 &= 1+\cos^2\eta_p+\cos^2\zeta_p+2\cos\eta_p+2\cos\zeta_p \\ &+ 2\cos\eta_p\cos\zeta_p+\sin^2\eta_p+\sin^2\zeta_p+2\sin\eta_p\sin\zeta_p\end{aligned} \quad (5)$$

which can be simplified to

$$\left|\widetilde{\psi}_p(\mathbf{k})\right|^2 = 3+2\left(\cos\eta_p+\cos\zeta_p+2\cos\eta_p\cos\zeta_p\right) \quad (6)$$

The maximal value of this expression is achieved when both $\eta_p = 2\pi\nu$ and $\zeta_p = 2\pi\mu$, where ν and μ are integer numbers. From the latter, taking for example $\nu = 0$, it is easy to obtain, for $p=1$, $k_y = K$ (where $K = 4\pi/3\sqrt{3}a$), and $k_x = 0$, and for $p=-1$, $k_y=-K$ and $k_x=0$.

Supplementary Note 2. Vortex velocity

We have studied how the vortex velocity depends on the parameters of the system in order to check that the propagation along the interface is not linked with the well-known vortex rolling effect. First, let us see that the vortex really follows the interface, and its core is located exactly within the unit cell, which separates the two inverted materials. Supplementary Figure 1 shows a snapshot of the phase of the wavefunction with a vortex. A 2π phase jump line is clearly visible, and the core of the vortex is located at the end of this line. The rotation direction of the vortex is shown with a red arrow, and the green arrow indicates the propagation direction of the vortex along the interface (white dashed line). We see that the edge of the phase jump line is within the unit cell located at the interface.

One might think that the vortex is simply rolling along the interface, like a wheel, converting rotation into propagation. The characteristic distance at which the density can vary in the condensate is given by the healing length ξ and therefore the center of the vortex in this "rolling wheel" image has to be located at a distance ξ from the wall, which allows to find the speed of rotation of the particles where they meet with the wall (and therefore the vortex propagation speed) using the expression

$$v = \frac{\hbar}{m}\frac{1}{r} \quad (7)$$

where one takes $r=\xi$, which gives simply that the vortex propagates with a velocity roughly equal to the speed of sound in the condensate $v=\sqrt{\alpha n/m}$. In this model, one could therefore expect a pronounced dependence of the vortex propagation velocity on the particle density. Another alternative could be that the vortex simply propagates with the group velocity of linear states at the interface, which can be calculated from the dispersion, as discussed in the main text. Supplementary Figure 2 compares the predictions of these models as a function of interaction energy αn with numerical results (black squares). Clearly, the simple predictions of the two naive

models (red circles for rolling effect and black dashed line for the linear group velocity) strongly deviate from numerics. The model of the rolling wheel (red dots) predicts a dependence on the density which is not observed at all (the interaction energy changes by a factor 5, and there is no significant change of the vortex velocity). The group velocity of the interface states strongly overestimates the real vortex propagation speed (also by a factor 5).

To calculate the vortex velocity, we analyze the currents that take place within its core (concentrated in a given valley because of winding-valley coupling). In the bulk, the valley states are not propagating, but rotating, because the 3 quantum-mechanical current terms between the 3 pillars of the same type which have different phases (0, $2\pi/3$, $4\pi/3$) exactly compensate each other, as these are three identical vectors rotated at 120 degrees. Indeed,

$$j = \frac{n\hbar}{m}\nabla\varphi \tag{8}$$

where n is the particle density, and therefore, to calculate current in the tight-binding approach we need to consider only pillars with nonzero density and take into account the phase difference between each pair.

At the interface the situation changes, as can be seen in Fig. 4(a) of the main text. The A pillars on the left of the interface are not large pillars (with lower energy) but small pillars (with higher energy), and therefore, the 3 current terms (blue arrows) do not have the same prefactor. The phase differences are the same, but the density on the pillars on the left of the interface is smaller (it is not zero as it would be in the bulk, because the presence of the interface mixes the Bloch states), and therefore the current term marked as a dashed line has a smaller magnitude than the other two. This results in a net current pointing upwards, and this is what leads to the propagative nature of the interface states.

The total current reads

$$\mathbf{j} = \mathbf{j}_1 + \mathbf{j}_2 + \mathbf{j}_3. \tag{9}$$

Assuming that the density on the A pillars on the right of the interface is n and the density on the A pillars on the left of the interface is n', we can write the magnitude of the current terms as:

$$j_{1,2} = \frac{n+n'}{2}\frac{\hbar}{m}\frac{2\pi}{3\sqrt{3}a} \tag{10}$$

and

$$j_3 = \frac{n'\hbar}{m}\frac{2\pi}{3\sqrt{3}a} \tag{11}$$

The orientation of the vectors makes that the X projection of j_3 is 0, while the X projections of j_1 and j_2 are opposite, and so they compensate each other. The Y projections give:

$$j_Y = \frac{1}{2}\left(j_1 + j_2\right) - j_3 \tag{12}$$

which finally gives

$$j_Y = \frac{n-n'}{2}\frac{\hbar}{m}\frac{2\pi}{3\sqrt{3}a} \tag{13}$$

Without the interface, $n = n'$ and $\mathbf{j} = 0$, as expected. The presence of the interface makes $n' < n$. If we consider an isolated problem of two pillars with coupling J and energy splitting Δ (which determines the gap in the bulk TMD analog), we can estimate n' as

$$n' = \frac{2n}{1 + \left(\Delta + \sqrt{\Delta^2 + 4J^2}\right)^2 / 4J^2} \tag{14}$$

which finally gives the expression for the group velocity of the main text, because $2\pi\hbar/m/3\sqrt{3}a$ is simply an estimate of the group velocity v_g in terms of the tight-binding parameters.

We can also calculate an approximated expression, assuming that $\Delta \ll J$,

$$n' = n\left(1 - \frac{\Delta}{2J}\right) \tag{15}$$

which gives for the net velocity along the interface

$$v_Y \approx \frac{\Delta}{2J}\frac{\hbar}{m}\frac{2\pi}{3\sqrt{3}a} \tag{16}$$

The corresponding calculated velocity shown in Supplementary Figure 2 by a solid black line corresponds well to the numerical results, contrary to the predictions of the simple models.

In the opposite limit of very large Δ,

$$n' = n\frac{2J^2}{\Delta^2} \tag{17}$$

and

$$v_Y \approx \left(1 - \frac{2J^2}{\Delta^2}\right)\frac{\hbar}{m}\frac{2\pi}{3\sqrt{3}a} \tag{18}$$

This expression also increases with the increase of Δ. It is interesting to see that this expression is bounded from above by a limiting value, which cannot be exceeded by changing Δ (but only by changing J, which affects m).

For a smooth interface, the difference between n and n' is strongly reduced and the vortex velocity drops down, limiting the possibilities of experimental observation and practical applications. The vortex also becomes less bound to the interface and its propagation can be easily perturbed by defects, because the topological protection, provided by the gap size, is reduced. The abrupt transition between the staggering domains therefore seems to be a better configuration.

Supplementary Note 3. Interface size: tight-binding study

In the main text, we argue that the interface can be made larger by inserting several zigzag chains of regular (non-staggered) honeycomb lattice between the two staggered domains. It is however important to check that the linear valley-polarized chiral interface states are still well defined in this situation. To elucidate this question, we consider ribbons of coupled infinite zigzag chains in the tight-binding approximation for several configurations.

The calculated projected dispersions are plotted in Supplementary Figure 3. Colors correspond to localization at the edge (blue) or at the center (red) of the ribbon. In panel (a), we plot the dispersion of 10 coupled regular zigzag chains ($\Delta = 0$) surrounded by vacuum. This is not an interface, but just a plain graphene ribbon. As expected, the dispersion is gapless, with flat edge states connecting the two valleys. For other panels, this ribbon is inserted between two staggered honeycomb lattices. For this, we consider a ribbon of a total number of 64 coupled zigzag chains with different domains inside. In panels (b)-(c), the interface contains 10 zigzag chains with $\Delta = 0$, whereas the domains on both side are staggered ($\Delta = \pm 0.25J$) with the same (b) or opposite (c) staggering potential . In the case where the staggering is the same on both sides, the system is fully isolating, even in the central regular honeycomb region (red curves), where a gap is opened due to the coupling of the graphene stripe to the two staggered domains. In the case where we have opposite staggering on each side of the interface (panel (c)) the chiral modes crossing the gap are still present. This means that the domain wall topological invariant $N_{K,K'} = \pm 1$ defined in the main text and the corresponding reasoning predicting the existence of the interface states is still valid when the interface width is increased. If we increase the interface region further, the energy bandgap in this central region remains open (except the single interface mode crossing it) and there is still a single mode crossing the gap in each valley, as predicted by the topological arguments. Finally, another example for an interface of 20 zigzag chains is plotted in panel (d).

Supplementary Note 4. Interface properties and disorder effects

As discussed in the main text, vortices stay attached to the interface thanks to the interplay of kinetic and interaction energy. The interface itself represents a barrier of kinetic energy for vortices. On the other hand, the presence of the interface modifies the density of the condensate which creates a potential trap for the vortices. The sum of the two forms a double-trap structure at both sides of the interface.

In the linear regime, a localized defect leads to an important scattering between valleys and perturbs the quantum valley Hall effect. Vortices are much less affected by such potential, because the intervalley scattering is suppressed for them due to valley-winding coupling. However, the vortices are vulnerable against tunneling in real space, between both sides of the barrier. Crossing the barrier means that the vortices start to propagate in the opposite direction. Vortex tunneling has been studied in the past since the discovery of magnetic vortices in superconductors. Two main mechanisms can be responsible for this tunneling: quantum-mechanical [1, 2] and thermal [3].

The quantum-mechanical tunneling rate is proportional to the amplitude of the pinning potential and exponentially decays with the square of the distance and with the density [1]: $t_{QM} = V_{pin}\exp(-\pi n d^2/2)$. For polaritons, the interaction constant [4] is $\alpha \approx 9$ μeV/μm^2 meaning that for the interaction energy of 0.3 meV the polariton density is of the order of 30 μm^{-2} and the corresponding tunneling rate for a tunneling distance of 1 μm is less than 10^{-11} s^{-1} (three thousand years), supposing a pinning potential V_{pin} of the order of 1 meV. This rate can therefore be made negligible if the width of the barrier is sufficiently large.

The thermal tunneling, responsible for the well-known vortex creep [3], is governed by the thermal activation mechanism involving an exponent of the barrier height $\sim \exp(-\Delta/k_B T)$. If one considers the specific example

of polaritons, they are strongly decoupled from the thermal reservoir thanks to their photonic fraction and the steepness of their dispersion. Thermal broadening in polariton systems like polariton graphene is comparable with the broadening induced by the lifetime [5]: the total broadening σ is of the order of 30 μeV, and the condition for the observation of both linear and non-linear effects linked with the interface is essentially the same: $\sigma < \Delta$, as stated in the main text. In our simulations, stable vortex propagation on one side of the interface is observed despite the residual bogolons (density waves) in the condensate that can be seen in the Supplementary Movies.

Even though the quantum and thermal tunneling are suppressed, a localized defect can perturb the interface barrier. While positive potential barriers repel vortices and do not lead to the crossing of the interface, negative potential traps can partially destroy the protective effect of the interface and make a vortex to cross it. Our simulations show that this is indeed possible, if the size of the defect is comparable with the size of a single pillar (or larger), while its amplitude is comparable with the total size of the conduction band ($6J \propto 1$ meV), much higher than the size of the gap (0.1 meV).

However, as shown in the previous section, the interface width can actually be increased by inserting several regular (not staggered) zigzag chains without affecting the behavior of the chiral interface states. The thickness of the barrier leads to an exponential decrease of the vortex tunneling probability and also enhances protection against potential defects, ensuring that the vortices always remain at the same side of the interface. In this sense, the interface is similar to the bulk of the sample: there is an exponentially small overlap of the edge states, which decreases with the increase of the sample size (for the bulk) or of the interface thickness (for the interface), and which provides protection against scattering on relatively large defects, which for the bulk would lead to scattering to the opposite side of the sample, and for the interface would lead to scattering to the opposite side of the interface.

We have checked that the vortex propagation is robust against defects up to ± 1.5 meV ($\sim 6J$, the size of the whole band) in amplitude and up to 2.5 μm ($\sim$lattice constant) in size. Defects with the amplitude of the order of the gap do not perturb the vortex propagation even with larger sizes (checked up to 5 μm).

As discussed in the main text, a single-domain sample can also possess QVH edge states with a non-zero group velocity. While this configuration leads to a non-zero velocity of vortices along the interface (inherited from the linear states via the vortex energy dependence on the wavevector $E_v(k_0)$, it does not provide the same double-well localization potential for vortices $E_v(x_0)$ as the one in Fig. 2(c) of the main text (see Supplementary Figure 4, black solid line). Indeed, if the flake is surrounded by vacuum (zero condensate density), the transverse potential (roughly given by the condensate density profile) exhibits a steady decrease across the interface (red dashed line), which means that a vortex can escape from the flake into the zero-density region and disappear. This is shown in Supplementary Movie 7. If the flake is surrounded by a constant-density condensate, the potential is modified in the opposite way: the vortex is repelled from the interface into the bulk (blue dotted line, see also Supplementary Movie 8). In both cases, edge states for vortices with an energy corresponding to the bulk gap (for vortices) do not exist anymore. The two-domain structure with an abrupt domain wall represents therefore the optimal configuration for the observation of the QSH analog for vortices.

Supplementary References

[1] Auerbach, A., Arovas, D. P. & Ghosh, S. Quantum tunneling of vortices in two-dimensional condensates. *Phys. Rev. B* **74**, 064511 (2006). URL `https://link.aps.org/doi/10.1103/PhysRevB.74.064511`.

[2] Fialko, O., Bradley, A. S. & Brand, J. Quantum tunneling of a vortex between two pinning potentials. *Phys. Rev. Lett.* **108**, 015301 (2012). URL `https://link.aps.org/doi/10.1103/PhysRevLett.108.015301`.

[3] Anderson, P. W. Theory of flux creep in hard superconductors. *Phys. Rev. Lett.* **9**, 309–311 (1962). URL `https://link.aps.org/doi/10.1103/PhysRevLett.9.309`.

[4] Ferrier, L. *et al.* Interactions in confined polariton condensates. *Phys. Rev. Lett.* **106**, 126401 (2011). URL `https://link.aps.org/doi/10.1103/PhysRevLett.106.126401`.

[5] Jacqmin, T. *et al.* Direct observation of dirac cones and a flatband in a honeycomb lattice for polaritons. *Phys. Rev. Lett.* **112**, 116402 (2014). URL `http://link.aps.org/doi/10.1103/PhysRevLett.112.116402`.
