# Supplementary material for: Robust quantum valley Hall effect for vortices in an interacting bosonic quantum fluid
Source: Nat Commun. 2018 Sep 28;9:3991. doi: 10.1038/s41467-018-06520-7 (PMC6162209; doi:10.1038/s41467-018-06520-7)
Supplement: Supplementary file 3 — Description of Additional Supplementary Files [file 41467_2018_6520_MOESM3_ESM.pdf]

The legends for the Supplementary Movie files:

Supplementary Movie 1. Propagation of a Gaussian wave packet, centred on one valley, in the linear regime in presence of the same defect as the one acting on the quantum vortex (1 meV and 1 µm size). A very significant fraction of the wave packet is scattered backwards.

Supplementary Movie 2. Temporal evolution of the spatial density distribution of the condensate, obtained by direct solution of the Gross-Pitaevskii equation without the tight-binding approximation. The snapshots from this movie are shown in Fig. 3 of the main text. The vortex is attached to one side of the interface and propagates along it, passing around two corners and a defect.

Supplementary Movie 3. Propagation of a vortex in a system with a wide (non-staggered) interface, as discussed Supplementary Notes 4, 5. This simulation does not include any defects.

Supplementary Movie 4. Propagation of a valley-polarized wave packet in the linear regime along the wide interface completely free of defects shows that there is no difference with a narrow interface.

Supplementary Movie 5. Behaviour of vortices in presence of a negative defect of 1 µm size and 1 meV amplitude (much larger than the gap) located at the wide interface. The density of the condensate is strongly perturbed by such a defect. Without the wide interface, the vortex would tunnel to the other side of the interface, but the wide interface prevents it, and the vortex continues to propagate in the same direction. The size of the interface should be chosen to be larger than the size of typical defects. Since those can be expected to be smaller than the size of a pillar, adding 1-2 extra zigzag chains without staggering should be sufficient.

Supplementary Movie 6. Propagation of a linear wave packet along a wide interface with a defect. The movie shows that the wide interface does not protect the linear states from scattering: a barrier of 1 meV and 1 µm in size still scatters approximately half of the wave packet to the other valley.

Supplementary Movie 7. Propagation of a vortex in a single-domain sample (with an additional potential on the edge, providing QVH edge states) *surrounded by vacuum*. The vortex is not protected from going into the zero-density region and disappearing. The position of the vortex is marked with a white cross.

Supplementary Movie 8. Propagation of a vortex in a single-domain sample (with an additional potential on the edge, providing QVH edge states), *surrounded by a constant density condensate*. The vortex is repelled from the interface into the bulk.

Supplementary Movie 9. A linear Gauss-Laguerre wave packet with a non-zero angular momentum does not at all exhibit the same behaviour as the vortex in an interacting condensate: the wave packet is unstable and expands rapidly, preventing the observer to keep trace of the propagation of its centre. The features of the interacting BEC maintaining the vortex are therefore crucial for the results obtained in the main text.
